# Metabolomics Reveals Nutritional Diversity among Six Coarse Cereals and Antioxidant Activity Analysis of Grain Sorghum and Sweet Sorghum

**DOI:** 10.3390/antiox11101984

**Published:** 2022-10-05

**Authors:** Yao Zhao, Guowei Zhai, Xuetong Li, Han Tao, Linying Li, Yuqing He, Xueying Zhang, Fulin Wang, Gaojie Hong, Ying Zhu

**Affiliations:** 1 State Key Laboratory for Managing Biotic and Chemical Treats to the Quality and Safety of Agro-Products, Institute of Virology and Biotechnology, Zhejiang Academy of Agricultural Sciences, Hangzhou 310021, China; 2 Key Laboratory of Biotechnology in Plant Protection of Ministry of Agriculture and Rural Affairs, Key Laboratory of Biotechnology in Plant Protection of Zhejiang Province, Hangzhou 310021, China; 3 Central Laboratory, State Key Laboratory for Managing Biotic and Chemical Threats to the Quality and Safety of Agro-Products, Zhejiang Academy of Agricultural Sciences, Hangzhou 310021, China

**Keywords:** coarse cereals, widely targeted metabolome analysis, grain sorghum, sweet sorghum, flavonoids, antioxidant activity

## Abstract

Coarse cereals are rich in dietary fiber, B vitamins, minerals, secondary metabolites, and other bioactive components, which exert numerous health benefits. To better understand the diversity of metabolites in different coarse cereals, we performed widely targeted metabolic profiling analyses of six popular coarse cereals, millet, coix, buckwheat, quinoa, oat, and grain sorghum, of which 768 metabolites are identified. Moreover, quinoa and buckwheat showed significantly different metabolomic profiles compared with other coarse cereals. Analysis of the accumulation patterns of common nutritional metabolites among six coarse cereals, we found that the accumulation of carbohydrates follows a conserved pattern in the six coarse cereals, while those of amino acids, vitamins, flavonoids, and lipids were complementary. Furthermore, the species-specific metabolites in each coarse cereal were identified, and the neighbor-joining tree for the six coarse cereals was constructed based on the metabolome data. Since sorghum contains more species-specific metabolites and occupies a unique position on the neighbor-joining tree, the metabolite differences between grain sorghum 654 and sweet sorghum LTR108 were finally compared specifically, revealing that LTR108 contained more flavonoids and had higher antioxidant activity than 654. Our work supports an overview understanding of nutrient value in different coarse cereals, which provides the metabolomic evidence for the healthy diet. Additionally, the superior antioxidant activity of sweet sorghum provides clues for its targeted uses.

## 1. Introduction

Coarse cereals generally refer to grain and legume crops except for rice and wheat, which have a small planting area, low yield, strong regionalism, and diverse cultivation methods compared to other major grains [1,2]. Coarse cereals include millet (*Setaria italica*), coix (*Semen coicis*), buckwheat (*Fagopyrum esculentum*), quinoa (*Chenopodium quinoa*), oat (*Avena sativa*), sorghum (*Sorghum bicolor*), maize (*Zea mays*), barley (*Hordeum vulgare*), etc. Coarse cereals are an important group of crops that constitutes staple food for a large number of people living primarily in the arid and semi-arid regions of the world [3]. Among them, sorghum has the characteristic of fast growth and high yields, and has been widely studied for its outstanding ability to adapt to various environments such as drought, high altitudes, saline-alkaline conditions, and barren soil [4,5]. Depending on its different uses, sorghum can be divided into grain sorghum and sweet sorghum. Grain sorghum has a high yield and is used for food and wine production, while sweet sorghum has a relatively low yield and large amounts of sugar (glucose, sucrose, fructose) accumulated in its stems and leaves, so it is used for sugar production, animal feed, and bioenergy [6].

As people’s desire for health and the demand for food increases, coarse cereals have become popular due to their comprehensive and high nutritional value. Coarse cereals are rich in dietary fiber, carbohydrates, minerals (especially micronutrients such as iron and zinc), amino acids, B vitamins, and secondary metabolites, giving coarse cereals many therapeutic effects [7]. For example, it has been shown that coarse cereals can regulate gut flora, and also contribute to alleviating cardiovascular diseases, neurodegenerative diseases, diabetes, obesity, and many cancers [8,9]. However, different coarse cereals exhibit varied health benefits due to their inclusion of differentiated metabolites. The phytochemicals in millet have the ability to decrease cholesterol and phytate levels in the human body [10]. Many in vivo studies have demonstrated that coix possess the capacity of anti-cancer, lipid-profile regulation, lower glycemic index, and anti-obesity properties [11]. The studies reported in [12,13] also reported that both oat and buckwheat (*F. esculentum*) have positive anti-inflammatory, anti-cancer, and antiatherogenic effects. Moreover, quinoa has also been found to contribute to metabolic, cardiovascular, and gastrointestinal health in humans [14]. Sorghum also has potential health benefits such as antioxidant activity, anti-diabetes, dyslipidemia, and cardiovascular diseases prevention [15]. However, although the benefits of various coarse cereals have been addressed in above studies, the differences in the metabolic profiles of these coarse cereals are not clear, and there is a lack of a targeted and comprehensive comparison among them.

Polyphenols are important components of coarse cereal metabolites in promoting human health. Polyphenols exist abundantly in coarse cereals, among which flavonoids account for the greatest proportion and are the most well studied [16]. Flavonoids are located in the pericarp of coarse cereals, and refer to a series of compounds in which two benzene rings are interconnected by three carbon atoms (C6-C3-C6), and contain several subclasses, such as flavones, flavonols, isoflavones, anthocyanins [16]. The flavonoid biosynthesis pathway in plants starts from phenylalanine, which is catalyzed by a series of enzymes to produce different kinds of flavonoids [17]. Some flavonoids are ingested into the body and metabolized by phase II enzymes of the small intestine into the systemic circulation [18]. Large amounts of flavonoids enter the large intestine and are converted into easily absorbed molecules by the gut flora [19]. A lot of studies have demonstrated that flavonoids are beneficial to human health. For example, flavonoids have been verified to reduce the risk of cardiovascular diseases, and type 2 diabetes. Meanwhile, the free radical scavenging and antioxidant properties of flavonoids have also been shown to reduce neurodegenerative diseases [20,21]. Therefore, the study of metabolites in coarse cereals, especially flavonoids, is important for understanding the nutritional value of coarse cereals.

In this study, the metabolic accumulation profiles of six types of coarse cereals, including millet, grain sorghum, oat, coix, buckwheat and quinoa, are compared to show the differences among them. Furthermore, the metabolome differences between grain sorghum and sweet sorghum are deeply analyzed, in which total flavonoid content and antioxidant activity were detected and compared. The results of this study provide a reference for healthy diet and a clues for understanding the targeted uses of the two kinds of sorghum.

## 2. Materials and Methods

### 2.1. Plant Materials

To study the diversity of metabolites in coarse cereals, six popular coarse cereals—millet (Qinzhouhuang, produced in Qinxian, China), coix (Xinrenbaike, produced in Xingren County, Guizhou Province, the largest producer of coix in China), buckwheat (Wensha, produced in Chifeng, China), quinoa (Jingle quinoa, produced in Jingle, China), oat (Baiyou1, produced in Guyang, China), and sorghum (grain sorghum 654 and sweet sorghum LTR108 were both produced at Zhejiang Academy of Agricultural Sciences, Hangzhou, China)—were selected for metabolome analysis.

### 2.2. Sample Preparation and Extraction

The mature and shelled seeds of millet, coix, buckwheat, quinoa, oat, and sorghum were freeze-dried by vacuum freeze-dryer (Scientz-100F, Ningbo, China) and crushed by a mixer mill (MM 400, Retsch, Germany) with a zirconia bead for 1.5 min at 30 Hz. Then, 100 mg of lyophilized powder was dissolved with 1.2 mL 70% methanol solution, vortexing 30 s every 30 min for 6 times in total, and the sample was placed in a refrigerator at 4 °C overnight. Following centrifugation at 12,000 rpm for 10 min, the extracts were filtrated with a 0.22 μm pore size Nylon Syringe Filter (SCAA-104, ANPEL, Shanghai, China) before LC-MS/MS analysis.

### 2.3. High-Performance Liquid Chromatography Conditions

Widely targeted metabolic profiling analyses were performed with an LC-ESI-MS/MS system (HPLC, Shim-pack UFLC SHIMADZU CBM30A system; MS, Applied Biosystems 6500 Q TRAP; Shimadzu, Kyoto, Japan). The analytical conditions were as follows: HPLC: column, ACQUITY UPLC HSS T3 C18 (1.8 µm, 2.1 mm × 100 mm, Waters, Shanghai, China); solvent system, water (0.04% acetic acid): acetonitrile (0.04% acetic acid); gradient program, 95:5 *v/v* at 0 min, 5:95 *v/v* at 11.0 min, 5:95 *v/v* at 12.0 min, 95:5 *v/v* at 12.1 min, 95:5 *v/v* at 15.0 min; flow rate, 0.40 mL/min; temperature, 40 °C; injection volume, 2 μL. The effluent was alternatively connected to an ESI-triple quadrupole-linear ion trap (Q TRAP)-MS.

### 2.4. ESI-Q TRAP-MS/MS

Linear ion hydrazine-flight time (LIT) and triple quadrupole (QQQ) scans were acquired on an API 6500 Q TRAP LC/MS/MS System, equipped with an ESI Turbo Ion-Spray interface, operating in a positive ion mode and controlled by Analyst 1.6 software (AB Sciex, Shanghai, China). The ESI source operation parameters were as follows: ion source, turbo spray; source temperature, 500 °C; ion spray voltage (IS), 5500 V; ion source gas I (GSI), gas II (GSII), curtain gas (CUR) were set at 55, 60, and 25 psi, respectively; the collision gas (CAD) was high. Instrument tuning and mass calibration were performed with 10 and 100 μmol/L polypropylene glycol solutions in QQQ and LIT modes, respectively. QQQ scans were acquired as MRM experiments with collision gas (nitrogen) set to 5 psi. DP and CE for individual MRM transitions were done with further DP and CE optimization. A specific set of MRM transitions were monitored for each period according to the metabolites eluted within this period.

### 2.5. Identification of Metabolites

We used Analyst 1.6.1 software to analyze the mass spectrometry data. To produce a matrix containing less biased and redundant data, peaks were checked manually for signal/noise (S/N) > 10, and removed the redundant signals caused by different isotopes, in-source fragmentation, K^+^, Na^+^, and NH_4_^+^ adducts, and dimerization. Metabolites in the samples were identified by comparing accurate *m/z* values, retention times (RT), and fragmentation patterns in the self-built MWDB database (Metware Biotechnology Co., Ltd., Wuhan, China) and the public database (MassBank [22] and METLIN [23]).

### 2.6. Determination of Flavonoids and Anthocyanin Contents

The total flavonoid contents of two different sorghums were determined as described previously [24]. The seeds of sorghum 654 and LTR108 were ground to powder with a mortar and pestle, 0.01 g was weighed, 1 mL extraction buffer (60% ethanol) was added, and it was shaken for 2 h at 60 °C, and centrifuged at 10,000× *g* at 25 °C for 10 min. Taking the supernatant, NaNO_2_ solution was added, and it was left to stand for 6 min at 25 °C. AlCl_3_ solution was added, and it was left to stand for 6 min at 25 °C. NaOH solution was added, and it was left to stand for 15 min at 25 °C, and the absorbance at 510 nm was determined using a microplate reader (Infinite 200 Pro, Tecan, Switzerland).

Anthocyanin quantification was performed as described previously [25]. First, 0.1 g sorghum seed powder was weighed and incubated overnight in 300 µL of 0.1% HCl-methanol solution at 4 °C. Then, 200 µL distilled water and 500 µL chloroform were added, followed by centrifugation at 10,000× *g* at a temperature of 4 °C for 10 min. The supernatant was taken, and the absorbances at 530 nm and 657 nm were detected by a microplate reader. The amount of total anthocyanins was calculated by subtracting the absorbance at 657 nm from that at 530 nm.

### 2.7. Determination of Antioxidant System Activity

The total antioxidant capacity (T-AOC) of the two different sorghums was determined using the ferric reducing antioxidant power (FRAP) method. T-AOC was obtained by measuring the change of absorbance at 593 nm. The amount of antioxidant Trolox per gram of sample was used to express the T-AOC of the sample.

CAT (Catalase) activities were determined based on the ammonium molybdate method. CAT activity was determined by measuring the change of absorbance at 405 nm. One unit of CAT activity was defined as the amount of decomposed 1 µmol of H_2_O_2_ per second per g of sample.

SOD (superoxide dismutase) activities were determined using the nitrotetrazolium blue chloride (NBT) method. SOD activity was determined by measuring the change of absorbance at 560 nm. One unit of SOD activity was defined as the amount of enzyme required to cause 50% SOD inhibition per g of sample in 1 mL of the reaction solution.

POD (peroxidase) activity was determined using the H_2_O_2_ method. POD activity was obtained by measuring the change of absorbance at 470 nm. One unit of POD activity was defined as the A470 change per min was 0.01 per g of sample in 1 mL of the reaction solution.

All these antioxidant system activities were determined using commercial kits (Cominbio Co., Ltd., Suzhou, China). The absorbance of each reaction mixture was measured using a microplate reader.

### 2.8. Statistical Analysis

Unsupervised principal component analysis (PCA), hierarchical cluster analysis (HCA), volcano plot and differential metabolites screening were performed using the R package (http://www.r-project.org (accessed on 1 September 2022)). The criterion for significantly different metabolites was *p* < 0.05, VIP (variable importance in projection) ≥ 1, and an absolute Log_2_FC (fold change) ≥2 in the Student’s t-test. Metabolic pathways were analyzed using the KEGG database (https://www..genome.jp/kegg/ (accessed on 1 September 2022) [26]. Student’s t-test in was used GraphPad Prism (https://www.graphpad.com/ (accessed on 1 September 2022) to compare bioactive substances and antioxidant system activity indexes of two different sorghums, in which significant differences between two groups are indicated by asterisks (*). The number of asterisks represents the degree of difference.

## 3. Results

### 3.1. Whole Metabolome-Scale Comparative Analysis of Six Coarse Cereals

**Figure 1:**
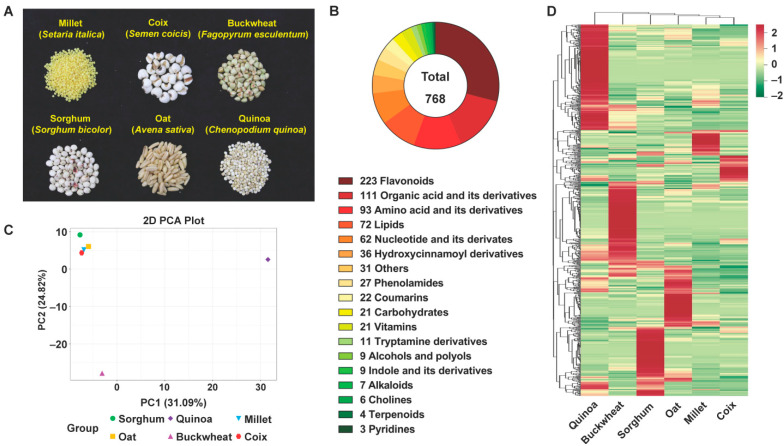
Metabolite profiles of six coarse cereals. (**A**) Photographs displaying six selected coarse cereals. (**B**) Classification of the 768 metabolites of six coarse cereals. (**C**) Principal component analysis (PCA) of metabolic profiles of six coarse cereals. (**D**) Heat map based on metabolome data of six coarse cereals. The content value of each metabolite was normalized, and hierarchical cluster analysis (HCA) was performed. Each coarse cereal species is represented by a column, and each metabolite is displayed in a row. Red shows relatively high metabolite abundance, while green indicates relatively low abundance.

To examine the diversity of metabolites, six popular coarse cereals, as shown in Figure 1A, namely millet (*S. italica*), coix (*S. coicis*), buckwheat (*F. esculentum*), quinoa (*C. quinoa*), oat (*A. sativa*), and grain sorghum (*S. bicolor*), were selected to perform a metabolomic analysis. Then, UPLC-MS-based widely targeted metabolomics was carried out, in which 768 metabolites were detected (Table S1). As shown in Figure 1B, the 768 metabolites can be classified in detail according to their properties: 223 flavonoids, 111 organic acids and their derivatives, 93 amino acids and their derivatives, 72 lipids, 62 nucleotides and their derivatives, 36 hydroxycinnamoyl derivatives, 27 phenolamides, 27 coumarins, 21 carbohydrates, 21 vitamins, 11 tryptamine, 9 alcohols and polyols, 9 indole and its derivatives, 7 alkaloids, 6 cholines, 4 terpenoids, 3 pyridines, and 31 others. Therefore, it can be seen that flavonoids are the most abundant metabolites in the six coarse cereals, and will be focused on later in this study.

To further explore the diversity of nutrients between six coarse cereals, we analyzed metabolic profiling by principal component analysis (PCA), and hierarchical cluster analysis (HCA). According to the PCA, two principal components, PC1 and PC2, represented a 31.09% and 24.82% contribution to the differences among six coarse cereals, respectively (Figure 1C). Furthermore, millet, coix, buckwheat, oat, and sorghum clustered together, quinoa is far from other grains in PC1. Meanwhile, buckwheat is separated from other grains in PC2. Therefore, the metabolite accumulation pattern in quinoa and buckwheat is quite different from the other four coarse cereals. The species-dependent accumulation pattern was further supported by a HCA based on coarse cereals metabolome (Figure 1D).

### 3.2. Comparative Analysis of Main Nutritional Metabolites in Six Coarse Cereals

**Figure 2:**
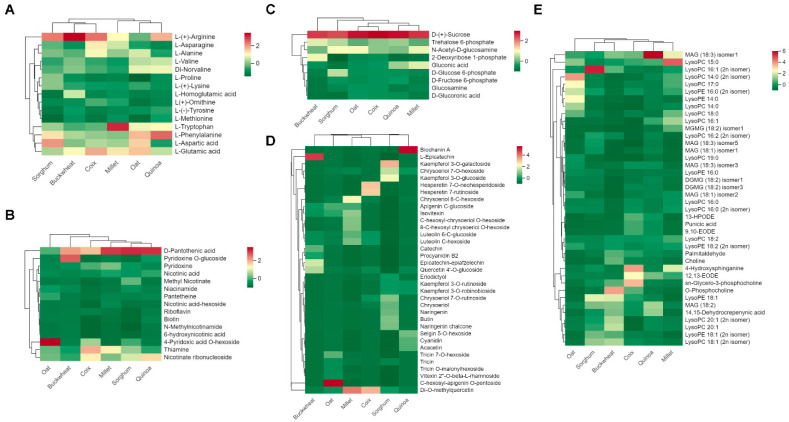
Accumulation of main nutritional metabolites in six coarse cereals. (**A**) Heat map of amino acids in six coarse cereals. (**B**) Heat map of vitamins in six coarse cereals. (**C**) Heat map of carbohydrates in six coarse cereals. (**D**) Heat map of flavonoids in six coarse cereals. (**E**) Heat map of lipids in six coarse cereals. The content value of each metabolite was normalized, and hierarchical cluster analysis (HCA) was performed. Each coarse cereal species is represented by a column, and each metabolite is displayed in a row. Red shows relatively high metabolite abundance, while green indicates relatively low abundance.

To illustrate the nutritional value of different coarse cereals, we compared the accumulation patterns of common nutritional metabolites amino acids, vitamins, carbohydrates, flavonoids, and lipids in six coarse cereals (Figure 2).

In addition to being the basic units of proteins, amino acids are also involved in signal transduction, redox balance, osmoregulation, and other life processes [27,28]. HCA of amino acid metabolome profiles showed that coarse cereals are rich in L-arginine, L-tryptophan, L-phenylalanine, L-aspartate, and L-glutamic acid, while other amino acids are relatively few (Figure 2A). On the other hand, the contents of L-arginine, L-tryptophan, L-phenylalanine, L-aspartate, and L-glutamic acid in the six coarse cereals are different. Sorghum, buckwheat, coix and quinoa all have higher L-arginine content, while millet and oats have lower L-arginine content. Millet contains a lot of L-tryptophan, and oats contain more L-phenylalanine, L-aspartate, and L-glutamic acid. In addition to L-arginine, sorghum is rich in L-aspartic acid, coix is rich in L-glutamic acid, and quinoa is rich in L-phenylalanine compared to other grains.

As organic compounds essential to life, vitamins play a vital role in life processes such as DNA synthesis, enzymatic reactions and oxygen transport [29,30]. It can be seen that vitamin B is very abundant in the six coarse cereals; in particular, vitamin B5 D-pantothenic acid is relatively high in the other five grains except for oat (Figure 2B). Oats and buckwheat are rich in vitamin B6, 4-pyridoxic acid O-hexoside and pyridoxine O-glucoside, respectively, both of which are vitamin B6. In addition, coix is rich in vitamin B1 thiamine, and quinoa is rich in vitamin B3 nicotinate ribonucleoside.

Carbohydrates, as the main energy source required by all living organisms to support their living activities, have conserved accumulation patterns in six coarse cereals (Figure 2C). Therefore, the main energy intake from consuming these six types of coarse cereals is the same. Further, three carbohydrates, D-(+)-sucrose, trehalose 6-phosphate, and N-acetyl-D-glucosamine are abundant in all six coarse cereals.

Plant flavonoids are beneficial to human health due to their biological activities, which include anti-oxidation, anti-bacteria, decreasing serum glucose and prevention of arteriosclerosis. As shown in Figure 2D, the flavonoids in the six coarse cereals were different and complementary. Specifically, buckwheat has more catechin derivative L-epicatechin than other coarse cereals, oats have more flavone C-glycoside C-hexosyl-apigenin O-pentoside than other coarse cereals, sorghum has more flavonol kaempferol 3-O-galactoside than other coarse cereals, quinoa has more isoflavone biochanin A than other coarse cereals. Both millet and coix have more flavonol di-O-methylquercetin than the other four coarse cereals, and coix is richer in flavanone hesperetin 7-O-neohesperidoside and hesperetin 7-rutinoside than the other five coarse cereals.

Lipids are important components of cell membranes, and can serve as fuel to drive energy-demanding processes and play a key role in numerous signal transduction processes [31]. Visualization of the lipids profile by HCA displayed apparent variation in their relative abundance in six coarse cereals (Figure 2E). Meanwhile, oat, sorghum, and millet all have higher lysoPC metabolites than other coarse cereals, in which lysoPC 17:0 (2n isomer) is contained in oat, lysoPC16:1 (2n isomer) is contained in sorghum, and lysoPC 15:0 is contained in millet. In addition, quinoa and buckwheat accumulated the most MAG (18:3) isomer1 and O-phosphocholine, respectively. Coix contains more 4-hydroxysphinganine and sn-glycero-3-phosphocholine than other coarse cereals.

### 3.3. Analysis of Species-Specific Metabolites and Evolutionary Relationships of Six Coarse Cereals

**Figure 3:**
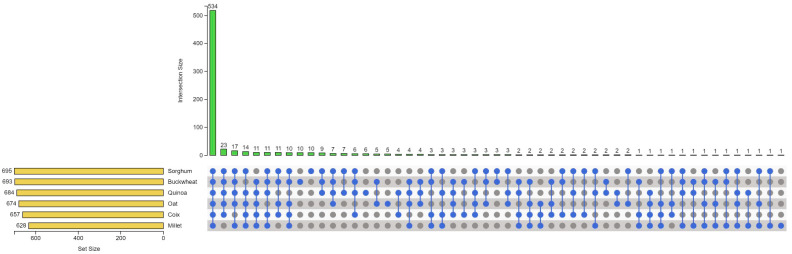
UpSet plot illustrating the overlapping and specific metabolites for six coarse cereals.

To show the remarkable species dependence of some of the metabolites in the coarse cereals, an UpSet plot was constructed as shown in Figure 3, in which there are 534 metabolites shared by six coarse cereals. It can be seen that metabolites exist in a single species: 10 in sorghum, 10 in buckwheat, 6 in quinoa, 5 in oat, and 1 in millet (Table S2). As shown in Table 1, four kinds of specific metabolites, namely six flavonoids, two coumarins, one terpenoid, and one quinate derivative, were contained only in sorghum. Ten species-specific metabolites, including four flavonoids, three quinate derivatives, one benzoic acid derivative, one coumarin, and one coumarin, were contained only in buckwheat. Moreover, one hydroxycinnamoyl derivative, four flavonoids, and one quinate derivative were contained only in quinoa. All five species-specific metabolites in oat were flavonoids, while the only species-specific metabolite in millet was also a flavonoid (7,4′-dihydroxyflavone).

**Figure 4:**
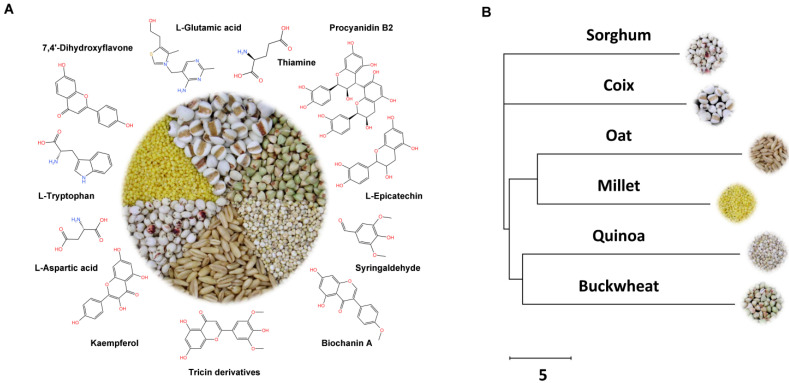
Analysis of species-specific metabolites and evolutionary relationships of six coarse cereals. (**A**) The representative species-specific metabolites or abundant metabolites among the six coarse cereals for each grain. (**B**) The neighbor-joining tree of the six coarse cereals.

As shown in Figure 4A, in combination with the data in Figure 2 and Table 1, the two most popular bioactive metabolites that were species-specific or most abundant for each grain were selected to analyze their physiological function in each coarse cereal. Considering the absence of species-specific metabolites in coix, L-glutamate and thiamine (vitamin B1), which have higher content in coix compared to in other grains, were selected to indicate the unique physiological function of coix. Further, although L-glutamate is a nonessential amino acid for the human body, it can also play a key role as a neurotransmitter in the human central nervous system in addition to its physiological functions such as preventing hair loss, enhancing blood circulation, and protecting the liver [32,33]. Thiamine is an essential vitamin for the production of adenosine triphosphate (ATP) in mitochondria, and a rate-limiting cofactor to multiple enzymes involved in the physiological processes [34]. Proanthocyanidins B2 and L-epicatechin are species-specific metabolites, and have more abundant metabolites than other coarse cereals in buckwheat. Both proanthocyanidins B2 and L-epicatechin are flavonoids, which have several therapeutic effects against different pathological states, including inflammation, cancer, cardiovascular and related diseases [35,36]. Species-specific metabolites of quinoa include syringaldehyde, which belongs to the phenolic aldehyde family and has anti-bacterial and anti-leukemic activities [37]. The relative content of isoflavone biochanin A (BCA) in quinoa is significantly higher than in other coarse cereals. BCA, in addition to the conventional effects of flavonoids, also has hepatoprotective, cardioprotective, osteoprotective, and neuroprotective effects [38]. The species-specific metabolites in oat are flavonoid tricin derivatives, which have anti-HIV, anti-leishmanial, anti-allergy, anti-obesity, and anti-UVB-induced damage bioactivities [39]. The species-specific metabolite in sorghum is kaempferol, and the representative metabolite that is more abundant than other coarse cereals is L-aspartic acid. Previous epidemiological research shows that kaempferol has therapeutic effects on bladder cancer, bone cancer, breast cancer, cervical cancer, colon cancer, gastric cancer, and other cancers [40]. Moreover, L-aspartic acid can be used as ammonia antidotes, liver function promoters, fatigue recovery agents, and other medical drugs. The only metabolite in millet not found in the other five coarse cereals is 7,4′-dihydroxyflavone. Recent studies have found that 7,4′-dihydroxyflavone acts as an aryl hydrocarbon receptor (AhR) antagonist in Caco2 cells [41]. The content of L-tryptophan in millet is much higher than that in other coarse cereals. Tryptophan is a precursor for the biosynthesis of many metabolites, such as serotonin, melatonin, and niacin, and is used to treat the depressive disorder, hypertension, vitamin B3 deficiency and other diseases [42].

Then, a neighbor-joining tree was constructed based on the metabolome data of the six coarse cereals. In a previous study, the neighbor-joining tree constructed from the metabolome data of ten fruits was compared with the evolutionary tree constructed from the single copy protein, and the results showed that the metabolomes reflected a close genetic relationship between different fruits to a certain extent [43]. As shown in the neighbor-joining tree (Figure 4B), quinoa and buckwheat are closely clustered, which are two grains with large differences in terms of metabolites from the other coarse cereals in Figure 1. Meanwhile, oats and millet, and sorghum and coix were also closely clustered, respectively. Sorghum and coix were progressively more distantly related to oats, millet, quinoa and buckwheat.

### 3.4. Analysis of Differential Metabolites and Antioxidant System Activities in Grain Sorghum and Sweet Sorghum

**Figure 5:**
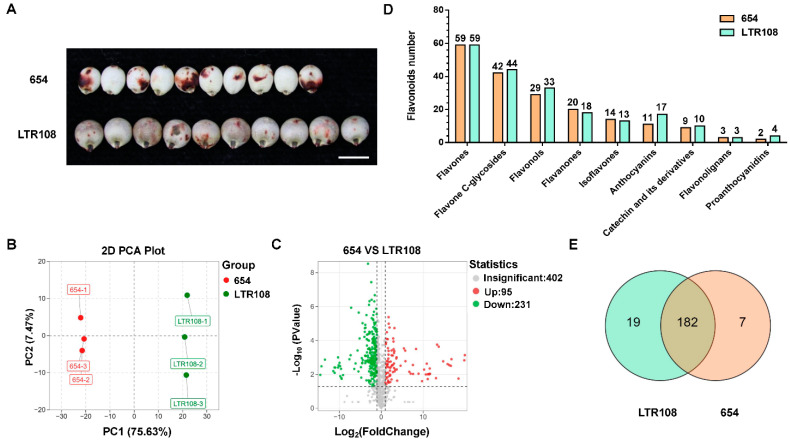
Differential metabolites analysis between grain sorghum and sweet sorghum. (**A**) Photographs displaying grain sorghum 654 and sweet sorghum LTR108 (Scale bar = 0.5 cm). (**B**) Principal component analysis (PCA) of metabolic profiles of 654 and LTR108. (**C**) Volcanic map analysis of differentially accumulated metabolites in 654 and LTR108. (**D**) Classification of the flavonoids of 654 and LTR108. (**E**) Venn diagram illustrating the overlapping and specific differential flavonoids for 654 and LTR108.

On the basis of metabolomic analysis, we found that grain sorghum 654 and buckwheat contained the most species-specific metabolites (Figure 3), and sorghum and coix were far away from the other four grains in the neighbor-joining tree (Figure 4B). Sorghum is widely cultivated worldwide due to its superior resilience under a variety of types of stress [44]. 654 and LTR108 are varieties of grain sorghum and sweet sorghum, respectively. 654 is the parent of many commercial sorghums in China with small-grains, while LTR108 is a large-grain sorghum (Figure 5A) [45,46]. To investigate the metabolic variation between grain sorghum and sweet sorghum, metabolomic analysis of 654 and LTR108 was performed (Table S3). PCA revealed that PC1 and PC2 explained 75.63% and 7.47% of the variability, respectively (Figure 5B). As shown in the PCA score plots, their metabolite accumulation profiles were different. Based on the OPLS-DA model, we screened the differential metabolites of 654 and LTR108. The screening results were presented as volcano plots, where 326 significantly different metabolites were screened, of which 95 were up-regulated and 231 were down-regulated (Figure 5C, Table S4). KEGG pathway analysis indicated that the differential metabolites were involved in 63 pathways (Table S5). Among them, the flavonoids biosynthesis pathway of secondary metabolites biosynthesis pathway has the most differential metabolites.

Flavonoid metabolites are widely found in plants, and have therapeutic effects on a variety of human diseases. Therefore, the flavonoid accumulation of 654 and LTR108, which contained 189 and 201 flavonoids, respectively, were analyzed in detail. As shown in Figure 5D, 654 contains 59 flavones, 42 Flavone C-glycosides, 29 flavonols, 20 flavanones, 14 isoflavones, 11 anthocyanins, 9 catechin and their derivatives, 3 flavonolignan and 2 proanthocyanidins. LTR108 contains 59 flavones, 44 Flavone C-glycosides, 33 flavonols, 18 flavanones, 13 isoflavones, 17 anthocyanins, 10 catechin and their derivatives, 3 flavonolignan and 4 proanthocyanidins. Further, it can be seen from the Venn diagram that 182 flavonoids are commonly contained in both 654 and LTR108, 7 flavonoids are contained only in 654, and 19 are contained only in LTR108 (Figure 5E). The species-specific flavonoids in 654 and LTR108 are shown in Table 2.

**Figure 6:**
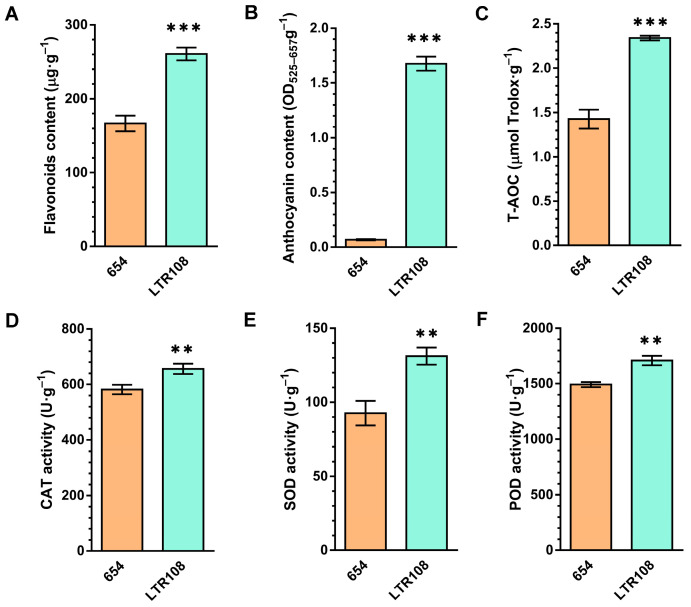
Bioactive substances and antioxidant system activity indexes of two different sorghums. Determination of total flavonoid content (**A**) and total anthocyanin content (**B**) in 654 and LTR108. Determination of total T-AOC (**C**), CAT activity (**D**), SOD activity (**E**) and POD activity (**F**) in 654 and LTR108. Bars indicate the s.d. of three replicates. ** *p* <  0.01, *** *p* < 0.005.

As can be seen from the above results, the variety of flavonoids in sweet sorghum LTR108 is slightly greater than in grain sorghum 654 (Figure 5D). We further detected the contents of total flavonoids (Figure 6A) and anthocyanins (including proanthocyanidins) (Figure 6B), and found that both of them were significantly higher in LTR108 than in 654. Considering the antioxidant activity of the flavonoids, the antioxidant system activity indexes of 654 and LTR108 were determined. It can be seen that the T-AOC of 654 was higher than that of LTR108 (Figure 6C). On the other hand, as three important active oxygen scavenging enzymes in plants, the activities of CAT, SOD, and POD were higher than those in 654 (Figure 6D–F), which was similar to T-AOC. In conclusion, flavonoids, especially anthocyanins, are more abundant in sweet sorghum LTR108 seeds than in grain sorghum 654, testifying to the antioxidant capacity of LTR108 seeds being better than that of 654 seeds.

## 4. Discussion

Due to the richness of various valuable nutrients, coarse cereals are playing an increasingly important role in the human diet and will gain more and more attention. The health benefits of each coarse cereal vary because of differences in metabolites. The metabolome has become an effective tool for analyzing metabolite accumulation patterns and has been used in the nutritional analysis of many grains. For example, a previous study used a widely targeted metabolomics-based approach to investigate the metabolite components of black, red, glutinous, and common white rice [47]. Another study used GC-MS (gas chromatography-mass spectrometry) to analyze phenolics and organic acids metabolomic profiling of wheat, barley, oat and rye, the main cereal crops in northern Europe [48]. In another work, the metabolic profiles of flavonoids and carotenoids were analyzed in 17 varieties of wheat, maize, rice, sorghum, foxtail millet, and broomcorn millet using the metabolome, and a total of 201 flavonoid metabolites and 29 carotenoid metabolites were dientified [49]. Meanwhile, they found that maize, sorghum, and foxtail millet grains were more abundant in flavonoids and carotenoids than other grains. However, a whole metabolome-scale comparative analysis among coarse cereals has yet to be performed. In this study, we performed widely targeted metabolic profiling analyses of six popular coarse cereals, millet, coix, buckwheat, quinoa, oat, and sorghum by UPLC-ESI-MS/MS (Figure 1), providing the scientific basis for the targeted diet. In addition, we further analyzed the differences in the main nutritional metabolites and species-specific metabolites in six coarse cereals, and found that the accumulation pattern of carbohydrates was conserved, while amino acids, vitamins, flavonoids and lipids were complementary (Figure 2). Therefore, the six coarse cereals involved in this study, despite having similar energy from carbohydrates, have more significant differences in terms of nutrient value, which allows for the targeted selection of relevant nutrients according to health requirements.

Sorghum is at the forefront of agriculture’s battle against climate change, and was domesticated 3000 to 5000 years ago and is now a resilient staple crop for 500 million people in Africa and Asia [4,5]. According to our results, sorghum contains many species-specific metabolites, which is far from other coarse cereals in terms of evolutionary relationships (Figure 3 and Figure 4B), so our further metabolomic study focused on sorghum. By comparing the metabolite differences between the two sorghums, we found that the content of flavonoids, especially anthocyanins, in sweet sorghum LTR108 was higher than in grain sorghum 654 (Figure 6). Moreover, the T-AOC and the activities of CAT, SOD and POD of LTR108 were also higher than those of 654 (Figure 6). Yan et al. also detected the activity of the POD enzyme in 654 and LTR108, which was the same as our results [50]. Additionally, it was found that the flavonoid content in rice seeds exhibited a positive correlation with grain length and length-to-width ratio [51]. Another author found that rice *GSA1* (*Grain Size and Abiotic stress tolerance 1*) could regulate auxin level and related gene expression through flavonoids, and finally regulated grain size by modulating cell proliferation and expansion [52]. The grain size of LTR108 is larger than 654 [46], probably because LTR108 contains a higher content of flavonoids. On the other hand, the current uses of sweet sorghum are based on the characteristics of high sugar content in its stems and leaves, such as sugar production and bioenergy. Considering the high flavonoid content and strong antioxidant activity of sweet sorghum seeds, food and wine with high nutritional value and unique flavor can be made.

## 5. Conclusions

In conclusion, this study compared the differences in the metabolic profiles of millet, coix, buckwheat, quinoa, oat, and sorghum by widely targeted metabolome analysis and found that quinoa and buckwheat showed significantly different metabolomic profiles than the other coarse cereals. Further, we analyzed the differences in main nutritional metabolites and species-specific metabolites in six coarse cereals, finding that grain sorghum and buckwheat contained the most species-specific metabolites. Additionally, a neighbor-joining tree for the six coarse cereals based on the metabolome data was constructed, which showed that grain sorghum and coix were far away from the other four grains. Finally, we compared the metabolite differences between grain sorghum and sweet sorghum, and detected the content of flavonoids and antioxidant activity. We found that LTR108 contained more flavonoids and had higher antioxidant activity than 654. Our work enriches knowledge of the metabolomics of coarse cereals, and provides metabolic evidence for a healthy diet and clues for the targeted uses of grain sorghum and sweet sorghum.

## Figures and Tables

**Table 1:** The species-specific metabolites found in six coarse cereals.

Species	ID	Q1 (Da)	RT (min)	Compounds	Class
Sorghum	pme0196	285	5.73	Kaempferol	Flavonol
	pme3288	329	6.63	3,7-Di-O-methylquercetin	Flavonol
	pme3300	301	4.51	Tricetin	Flavone
	pme1496	267.1	6.33	4′-O-methyldaidzein	Isoflavone
	pme3502	429.1	4.59	Formononetin 7-O-glucoside	Isoflavone
	pmb0631	581.1	3.17	C-hexosyl-luteolin C-pentoside	Flavone C-glycosides
	pmb4777	337.1	4.4	4-Hydroxy-7-methoxycoumarin-beta-rhamnoside	Coumarins
	pmb2601	307.1	307.1	7-hydroxycoumarin-beta-rhamnoside	Coumarins
	pme0062	515.3	5.88	Cucurbitacin D	Terpenoids
	pmb2833	529	2.13	3-O-Feruloyl quinic acid glucoside	Quinate derivatives
Quinoa	pmb2835	181.1	3.95	Syringaldehyde	Hydroxycinnamoyl derivatives
	pme3468	739.2	3.35	Kaempferol-3-O-robinoside-7-O-rhamnoside	Flavonol
	pme3399	445.1	5.16	Sissotrin	Isoflavone
	pme1773	595	2.7	Cyanidin 3-O-rutinoside	Anthocyanins
	pmb0542	535.1	2.99	Cyanidin 3-O-malonylhexoside	Anthocyanins
	pmb3061	499.1	2.1	5-O-p-coumaroyl quinic acid O-hexoside	Quinate derivatives
Millet	pme3507	253.1	4.75	7,4′-Dihydroxyflavone	Flavone
Oat	pme0434	653.1	4.16	Tricin di-O-hexoside	Flavone
	pmb3053	523.1	6	Tricin O-eudesmic acid	Flavone
	pmb3031	403.1	5.23	Tricin O-glycerol	Flavone
	pmb2979	549.2	3.91	Hesperetin O-malonylhexoside	Flavone
	pmb3052	525.1	5.84	Tricin 4′-O-β-guaiacylglycerol	Flavonolignan
Buckwheat	pme0434	577	3.03	Procyanidin B2	Proanthocyanidins
	pmb2976	755.1	3.74	Chrysoeriol C-pentosyl-O-hexosyl-O-hexoside	Flavone C-glycosides
	pmb2977	739.2	3.87	Chrysoeriol 8-C-pentosyl-O-rutinoside	Flavone C-glycosides
	pmb3047	657.1	4.50	Tricin 4′-O-(syringyl alcohol) ether 5-O-hexoside	Flavonolignan
	pmb3066	481.1	2.62	5-O-p-coumaroyl shikimic acid O-hexoside	Quinate derivatives
	pmb3064	499.2	2.50	3-O-p-coumaroyl quinic acid O-hexoside	Quinate derivatives
	pmb3058	369	2.16	Quinic acid O-glucuronic acid	Quinate derivatives
	pme1162	169	1.74	Gallic acid	Benzoic acid derivatives
	pmb2947	865.1	3.44	Catechin-catechin-catechin	Catechin derivatives
	pme3131	221.1	5.39	6,7-Dimethoxy-4-methylcoumarin	Coumarins

**Table 2:** The species-specific metabolites in 654 and LTR108.

Species	ID	Q1 (Da)	RT (min)	Compounds	Class
654	pma0825	503	5.24	Chrysin O-malonylhexoside	Flavone
	pmb0744	435.3	5.48	Tricin O-phenylformic acid	Flavone
	pma0787	551.1	4.63	Quercetin-3-(6′-malonyl)-Glucoside	Flavonol
	pme2984	593	5.09	Isosakuranetin-7-neohesperidoside	Flavanone
	pme3464	285.1	6.98	Isosakuranetin	Flavanone
	pmb0631	445	3.54	Glycitin	Isoflavone
	pmb2586	593.1	3.72	Gallocatechin-catechin	Catechin derivatives
LTR108	pme1773	595	2.7	Cyanidin 3-O-rutinoside	Anthocyanins
	pmb0542	535.1	2.99	Cyanidin 3-O-malonylhexoside	Anthocyanins
	pmb0557	621.1	3.26	Cyanidin O-malonyl-malonylhexoside	Anthocyanins
	pme0094	447.3	2.61	Cyanidin 3-O-glucoside	Anthocyanins
	pmb2959	489.1	2.94	Cyanidin O-acetylhexoside	Anthocyanins
	pmb0558	637.1	3.27	Delphinidin O-malonyl-malonylhexoside	Anthocyanins
	pmb0837	577.1	2.92	Procyanidin A3	Proanthocyanidins
	pme0434	577	3.03	Procyanidin B2	Proanthocyanidins
	pmb3044	653.1	4.16	Tricin di-O-hexoside	Flavone
	pmb2999	461.1	3.87	Chrysoeriol 5-O-hexoside	Flavone
	pmb3013	519.1	4.32	Isorhamnetin O-acetyl-hexoside	Flavonol
	pme1588	315.1	5.85	Isorhamnetin	Flavonol
	pme1478	317	4.7	Myricetin	Flavonol
	pmb3026	505.1	3.8	Quercetin O-acetylhexoside	Flavonol
	pmb0615	789.1	2.58	Hesperetin C-hexosyl-O-hexosyl-O-hexoside	Flavone C-glycosides
	pmb0691	757.1	3.09	Luteolin C-hexosyl-O-rhamnoside O-hexoside	Flavone C-glycosides
	pme0450	289	3.32	L-Epicatechin	Catechin derivatives
	pmb2947	865.1	3.44	Catechin-catechin-catechin	Catechin derivatives

## Data Availability

Data are contained within the article.

## Supplementary Materials

The following supporting information can be downloaded at: https://www.mdpi.com/article/10.3390/antiox11101984/s1, Table S1: Metabolite variation in six coarse cereals were detected by LC-MS-based widely targeted.; Table S2: Intersection of six coarse cereals metabolites; Table S3: Metabolite variation in 654 and LTR108 were detected by LC-MS-based widely targeted; Table S4: Differential metabolites between grain sorghum and sweet sorghum; Table S5: KEGG pathway analysis of differential metabolites for two kinds of sorghum.
